# Chimäre Antigenrezeptoren (CARs) in der Onkologie: eine Übersicht zu klinischer Anwendung und neuen Entwicklungen

**DOI:** 10.1007/s00103-020-03222-8

**Published:** 2020-10-06

**Authors:** Alexander Michels, Jessica Hartmann, Christian J. Buchholz

**Affiliations:** Paul-Ehrlich-Institut, Paul-Ehrlich-Str. 51–59, 63225 Langen, Deutschland

**Keywords:** Hämatologische Tumorerkrankungen, Immuntherapie, Genetisch modifizierte Zellen, ATMP, CAR, Hematologic malignancies, Immunotherapy, Genetically modified cells, ATMP, CAR

## Abstract

2018 erhielten 2 neuartige Krebstherapien auf Basis chimärer Antigenrezeptoren (CARs) die Marktzulassung in der Europäischen Union. Die Produkte, zunächst zugelassen für die Bekämpfung weit fortgeschrittener Leukämien bzw. Lymphome, erhielten nicht nur wegen ihrer neuen Wirkungsweise und Behandlungserfolge viel Aufmerksamkeit, sondern auch wegen ihrer teilweise gravierenden Nebenwirkungen sowie der wirtschaftlichen und logistischen Herausforderungen, die mit ihrer Herstellung verknüpft sind. Nun, fast 2 Jahre später, belegen Hunderte laufende klinische Studien das weltweite Bestreben, das Potenzial der CAR-Technologie voll zu erschließen. Dazu gilt es, die Effektivität der Behandlung bei verschiedenen Krankheitsbildern sicherzustellen, das Nebenwirkungsprofil zu verstehen und zu verbessern und die Herstellung des Zellpräparats robuster zu gestalten.

In diesem Artikel beschreiben wir die Studienlage sowie das Wirkprinzip von CAR-T- und -NK-Zellen. In jüngster Zeit hat eine Reihe von Studien in fortgeschrittenen Tiermodellen Einblicke in die potenziellen Ursachen schwerer Nebenwirkungen der CAR-Therapie ermöglicht. Wir fassen diese Ergebnisse zusammen und erläutern die Funktionsweise verfügbarer Tiermodelle. Zusätzlich zeigen wir mögliche weitere Ansätze auf, die momentanen Limitationen der Technologie zu umgehen und sie breit und sicher einsetzbar zu machen. Bisher als letztes Mittel bei sehr schweren Krankheitsverläufen eingesetzt, scheint die CAR-Therapie am Beginn einer Entwicklung hin zu einem neuen Konzept bei der Behandlung eines breiten Spektrums von hämatologischen und soliden Tumorerkrankungen zu stehen.

## Abstract (en)

In 2018, two novel cancer therapies based on chimeric antigen receptors (CARs) were granted marketing authorization in the European Union. Authorized for use against advanced lymphoma and/or leukemia, the products were at the center of international attention, not only due to their novel mode of action and their encouraging efficacy but also because of their sometimes severe side effects and the economic and logistic challenges posed by their manufacture. Now, almost two years later, hundreds of active clinical trials emphasize the global drive to harness the full potential of CAR technology.

In this article, we describe the mode of action of CAR T and CAR NK cells and review the clinical testing situation as well as early real-world data. In recent years, preclinical studies using advanced animal models have provided first insights into the mechanisms underlying the severe side effects of CAR T therapy. We summarize their results and describe the available models. Additionally, we discuss potential solutions to the hurdles currently limiting CAR technology. So far used as last-line treatment for patients with aggressive disease, CAR technology has the potential to become a new, broadly effective standard for tumor therapy.

## Einleitung

Krebserkrankungen waren 2017 mit über 200.000 Toten die zweithäufigste Todesursache in Deutschland [1]. Deshalb werden dringend neue oder effektivere Wege zur Behandlung von Krebserkrankungen benötigt. In den letzten Jahren gab es im Feld der Immunonkologie bemerkenswerte wissenschaftliche Fortschritte:

Im Gegensatz zu klassischen Krebstherapien wie Chemotherapie oder Bestrahlung macht man sich hier das körpereigene Immunsystem zunutze. Dieses erkennt von Natur aus atypische molekulare Signaturen auf Zellen und ist in der Lage, diese zu zerstören. Das gilt insbesondere für Infektionen, aber auch bei Tumorerkrankungen kann das Immunsystem manchmal das unkontrollierte Zellwachstum verhindern. Oftmals fehlt es hier allerdings an passenden molekularen Erkennungsstrukturen. Bei der Immuntherapie werden Immunantworten gegen Krebszellen durch gezielte Stimulation des patienteneigenen Immunsystems hervorgerufen. Dies kann etwa durch Antikörper, die bestimmte Oberflächenproteine auf Krebszellen erkennen (wie der Anti-CD20-Antikörper Rituximab [2]), oder durch Maskieren von immunmodulatorischen Strukturen (engl. Checkpoint Inhibitors) auf Immun- und/oder Krebszellen, wie z. B. PD‑1 oder CTLA‑4 [3], geschehen. Viel Aufmerksamkeit erhielt in den letzten 2 Jahren die Immuntherapie mit chimären Antigenrezeptoren (engl. Chimeric Antigen Receptors*, *CARs).

So konnten bemerkenswerte klinische Erfolge bei der Behandlung von Patienten mit hämatologischen Krebserkrankungen durch die Gabe von CAR-T-Zellen erzielt werden. Die CARs sind dabei gegen das B‑zellspezifische Antigen Cluster of Differentiation 19 (CD19) gerichtet. Dies führte 2018 zur Zulassung von zwei CAR-T-Zellprodukten in Europa, nämlich Kymriah (Tisagenlecleucel, Novartis) und Yescarta (Axicabtagene ciloleucel, Kite). Die positiven klinischen Befunde wurden jedoch von Berichten über schwere Nebenwirkungen begleitet. So gab es Fälle von Zytokinfreisetzungssyndrom (engl. Cytokine Release Syndrome, CRS) und Neurotoxizität, einige Patienten verstarben [4].

Inzwischen haben Studien mit spezialisierten Mausmodellen wertvolle Hinweise zum Zustandekommen von CRS und Neurotoxizität geliefert [5–8]. In diesem Übersichtsartikel besprechen wir die verwendeten Ansätze und ihre Implikationen für die CAR-Therapie. Des Weiteren zeigen wir mögliche Wege auf, die logistischen und wirtschaftlichen Hürden beim Einsatz von CAR-Therapien zu umgehen, z. B. durch eine Automatisierung der Herstellung von CAR-Zellen.

## Was sind CAR-Zellen? Wie werden sie erzeugt?

**Figure 1:**
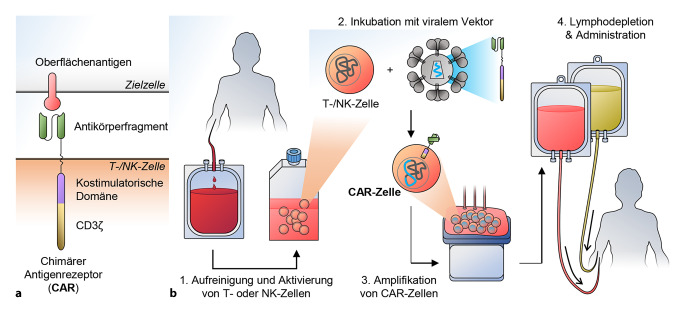

CARs sind aus mehreren Domänen verschiedener Immunrezeptoren zusammengesetzt, die einen synthetischen, chimären Rezeptor bilden (Abb. 1a). Ein Transmembranprotein mit einem einzigen Membrandurchgang präsentiert an *N*-terminaler Position die Antigenbindedomäne. Im Fall der zugelassenen CAR-Zellprodukte handelt es sich dabei um ein Antikörpereinzelkettenfragment (engl. Single Chain Variable Fragment, scFv), ein aus den variablen Teilen der schweren und leichten Kette eines Immunglobulins bestehendes Fusionsprotein, das die Antigenspezifität des CAR bestimmt. CARs können auch mit anderen Arten von Bindedomänen konstruiert werden, zum Beispiel solchen auf Basis von Nanobodies, Fab-Fragmenten oder Designed Ankyrin Repeat Proteins (DARPins; [9–11]). In C‑terminaler Richtung folgen auf die Antigenbindedomäne dann eine Scharnierregion (engl. Hinge) und die Transmembrandomäne. Obwohl die genauen Signaltransduktionsmechanismen von CARs noch nicht ausreichend verstanden sind, gibt es Hinweise auf die Wichtigkeit der Wahl von Scharnier- und Transmembrandomäne für eine optimale Signaltransduktion [10, 12]. Auf intrazellulärer Seite befinden sich Signaldomänen, die bei Bindung des Antigens das Signal weiterleiten. CARs der sog. zweiten Generation enthalten dabei die Signaldomäne des CD3-Komplexes (CD3ζ) sowie eine ein kostimulatorisches Signal auslösende Domäne, z. B. von CD28 oder 4‑1BB (CD137). Komplexere CARs können zusätzliche Signaldomänen („dritte Generation“) enthalten oder Strukturen, die ein Zytokinsignal hervorrufen („vierte Generation“, etwa TRUCKs; [4, 12, 13]).

CARs können sowohl in T‑Zellen als auch in natürliche Killerzellen (NK-Zellen) eingebracht werden und bewirken in diesen bei Antigenbindung eine Verschiebung der Signalverhältnisse zugunsten der Aktivierung der Zellen gegenüber Tumorzellen. Die Aktivierung von T‑Zellen ist, u. a. zur Vermeidung von Autoimmunreaktionen, ein streng regulierter Prozess. Wegen der subtilen bzw. transienten molekularen Unterschiede zwischen Krebszellen und gesunden Körperzellen und des Selbstursprungs von Tumorzellen kommt es häufig zu keiner (effizienten) Antitumorreaktion des Immunsystems. Kritische Komponenten der Aktivierung von T‑Zellen, sonst zwischen 2 Rezeptorkomplexen aufgeteilt, liegen bei CAR-T-Zellen unter der Kontrolle eines einzigen Signals, der Erkennung des Antigens durch die Antigenbindedomäne des CAR. So werden die kanonischen Immunsignalachsen, nämlich T‑Zellrezeptor und Kostimulation (z. B. CD28-CD80), die durch ihre komplexe Regulation Tumortoleranz begünstigen können, umgangen. Ein weiterer Unterschied zu normaler T‑Zellfunktion besteht in der Erkennung nativer Antigene durch den CAR, im Gegensatz zu proteolytisch prozessierten, durch Major Histocompatibility Complexes (MHCs) präsentierten Peptidantigenen, welche von T‑Zellrezeptoren erkannt werden [14].

Zusätzlich zu CAR-T-Zellprodukten werden aktuell auch auf NK-Zellen basierende Krebstherapien in klinischen Studien evaluiert. NK-Zellen agieren als Brücke zwischen angeborenem und adaptivem Immunsystem. Sie werden – im Gegensatz zu T‑Zellen – nicht über die MHC-TCR-Achse aktiviert, sondern integrieren Signale aus einem begrenzteren, in der Keimbahn codierten Repertoire polymorpher inhibitorischer und aktivierender Rezeptoren [15]. Erste klinische Studien haben gezeigt, dass nur teilweise mit Empfänger-MHC kompatible, allogene NK-Zellen in Kombination mit lymphodepletierender Vorbehandlung sicher und effektiv zur Behandlung von Tumorerkrankungen eingesetzt werden können [16]. Sie sind außerdem interessant für CAR-Therapien, weil sie zytotoxisch wirken und dabei CAR-mediierte und CAR-unabhängige Antitumoraktivität zeigen können. NK-Zellen sind mit den gleichen CAR-Architekturen kompatibel wie T‑Zellen.

Wenngleich die Prozesse zur Herstellung von in klinischen Studien eingesetzten CAR-Zellprodukten im Detail recht heterogen sind, gleichen sie sich in ihren grundlegenden Schritten. Die zur Herstellung eines autologen CAR-Therapieprodukts notwendigen Arbeitsschritte sind in Abb. 1b illustriert. Die relevanten Immunzellen (T- oder NK-Zellen) werden aus dem Blut des Patienten isoliert und außerhalb des Körpers (*ex vivo*) kultiviert. Zu geeigneten Zeitpunkten werden das Wachstum und Überleben der Zellen begünstigende Biomoleküle (wie agonistische Antikörper, Interleukine) zugegeben. Darauffolgend wird das für den CAR codierende genetische Material in die Zellen eingebracht. Meistens werden dafür retrovirale oder vom humanen Immundefizienzvirus 1 (HIV-1) abgeleitete lentivirale Vektoren verwendet. Retro- und lentivirale Vektoren integrieren die CAR-enthaltende Genkassette in das chromosomale Material der Zellen. Die resultierenden CAR-Zellen werden dann für einige Tage in Zellkultur amplifiziert, bevor das Zellmaterial, meist nach einer vorbereitenden Behandlung des Patienten mit Chemotherapeutika, reinfundiert wird [17, 18].

## CAR-Zellen in der klinischen Anwendung

Die beiden zugelassenen Arzneimittel, Axicabtagene ciloleucel und Tisagenlecleucel, sind derzeit die am häufigsten in der Klinik angewendeten CAR-T-Zellprodukte. In der für die Zulassung ausschlaggebenden Studie ZUMA‑1 zur Behandlung von aggressivem Non-Hodgkin-Lymphom in Patienten, bei denen Standardtherapien versagt hatten, erzielte Axicabtagene ciloleucel eine mit 66 % mehr als doppelt so hohe Ansprechrate wie bei der historischen Kontrolle [19]. Für die Indikation akute lymphatische Leukämie (ALL) erreichten in der zulassungsrelevanten Studie von 75 mit Tisagenlecleucel behandelten Probanden über 80 % vollständige Remission und über 75 % überlebten für mindestens 12 Monate nach der Behandlung. Beide Ergebnisse waren besser als die der herangezogenen Chemotherapie- und Antikörpervergleichstherapien [20]. Weniger eindeutig waren die Daten zur Behandlung des diffusen großzelligen B‑Zell-Lymphoms (DLBCL). Aufgrund der langen Zeit zwischen Patienteneinschluss und CAR-T-Zellinjektion (54 Tage) kam es hier zu einem ungewöhnlich hohen Anteil an Patienten, welche die Teilnahme an der Studie abbrachen. Ausschlaggebend für das positive Votum bei der Europäischen Arzneimittel-Agentur (EMA) waren dann u. a. die lang anhaltenden Effekte bei den Patienten, bei denen die Therapie angeschlagen hatte [20].

Seit den Zulassungserteilungen sind in der EU bis September 2019 mehr als 100 Patienten mit den kommerziellen Produkten behandelt worden [21]. Alle Patienten werden im Melderegister der *European Society for Blood and Marrow Transplantation* dokumentiert (www.EBMT.org). Außerdem erfolgen Nachbeobachtungsstudien, mit denen bei der Zulassung erhaltene Auflagen erfüllt werden. Sobald vollständig, werden die nach Zulassung gewonnenen Wirksamkeitsdaten interessante Vergleiche mit den zur Zulassung vorgelegten Daten ermöglichen. Erste Analysen für Axicabtagene ciloleucel aus dem kommerziellen Anwendungsbetrieb in der Behandlung von Non-Hodgkin-Lymphomen [22, 23] zeigen im Vergleich mit der ZUMA‑1 Studie ein geringfügig schlechteres Ansprechen [24]. Allerdings werden hier insbesondere die Langzeiteffekte zu bewerten sein, zu denen derzeit noch keine abschließenden Aussagen getroffen werden können [21].

**Figure 2:**
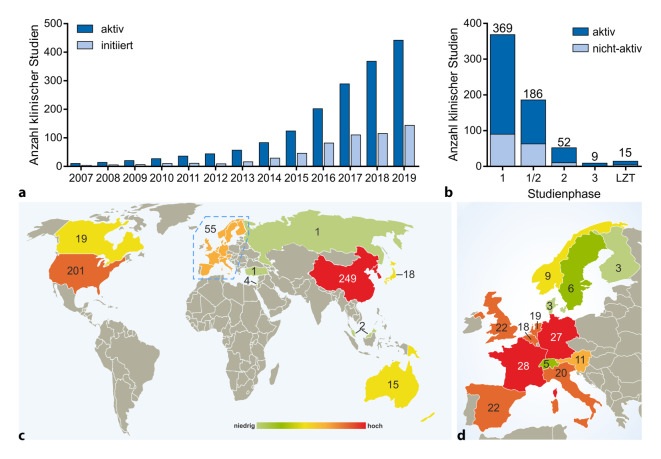

Neben der klinischen Anwendung der zugelassenen CAR-T-Zellen finden weltweit viele klinische Studien statt, in denen neuentwickelte CARs getestet oder bereits zugelassene unter neuen Bedingungen eingesetzt werden. Auf Basis des freiwilligen US-Registers *clinicaltrials.gov *[25] lässt sich die Dynamik der Studienlage analysieren. Zum Anfang des zweiten Quartals 2020 sind 675 CAR-T-Studien für Tumortherapien registriert, darunter 15 Langzeitstudien. Demnach stieg die Zahl aktiver Studien seit 2016 um mehr als 100 % auf 495 Studien weltweit, darunter 11 Langzeitstudien. 92 Studien sind bereits beendet. Die Anzahl aktiver Studien steigt also seit 2014 kontinuierlich an und hat sich seitdem knapp verfünffacht (Abb. 2a).

Die meisten CAR-T-Studien laufen in China und den USA (Abb. 2c). Seit 2017 hat sich die Zahl klinischer Studien dort etwa verfünffacht bzw. verdoppelt. Weniger als 10 % (36 von 495 Studien) der weltweit laufenden CAR-T-Studien sind multinational angelegt. Dies gilt insbesondere für Europa, wo an mehr als der Hälfte (29 von 55) der laufenden Studien mehrere Länder beteiligt sind. Dabei ist die gesamte Zahl laufender CAR-T-Zellstudien in Deutschland seit 2017 fast um den Faktor 10 gestiegen. Deutschland und Frankreich sind innerhalb der Europäischen Union führend an CAR-T-Studien beteiligt (27 bzw. 28 laufende Studien; Abb. 2c,d ). Weltweit befinden sich mehr als die Hälfte der registrierten Studien in Phase 1, inzwischen aber auch fast 30 der über 650 registrierten Studien in Phase 3 oder Langzeituntersuchungen (Abb. 2b). Letztere sind von großer Bedeutung, um besser zu verstehen, wie lange sich CAR-T-Zellen im Patienten aufhalten, dies sowohl im Hinblick auf etwaige Nebenwirkungen als auch auf ihre antitumorale Aktivität.

**Figure 3:**
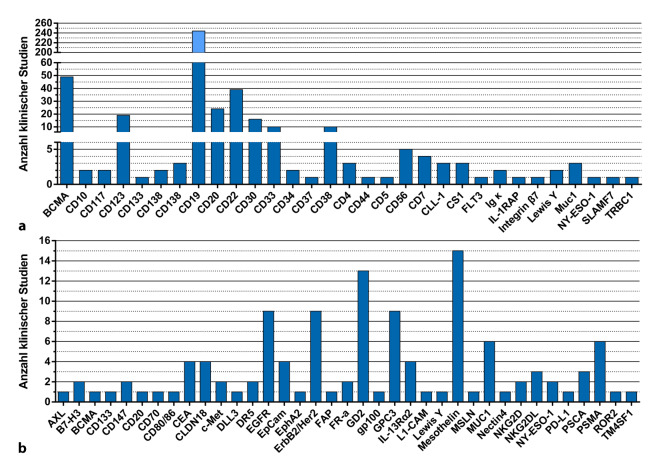

Die mit Abstand größte Anzahl klinischer Studien beruht auf CD19 als Zielantigen (Abb. 3). Dies mag verwundern, da doch bereits 2 solcher CAR-T-Zellprodukte über eine Marktzulassung verfügen. Genau hier zeigt sich allerdings das Potenzial dieses therapeutischen Ansatzes, welches mit den beiden zugelassenen Arzneimitteln selbst für CD19 als Zielantigen noch lange nicht erschöpft ist. So stehen in laufenden Studien mit CD19-CAR-T-Zellen Fragen im Fokus, welche die Ausweitung der Indikationen, Vereinfachungen im Herstellungsprozess, Veränderungen im CAR-Design, die Art der verwendeten T‑Zellsubtypen oder die Kombination verschiedener CARs mit Spezifität gegen mehrere Tumorantigene in einem Produkt betreffen.

Daneben werden in vielen laufenden Studien CAR-T-Zellen mit Spezifität für andere Zielmoleküle evaluiert. Dies betrifft nach wie vor insbesondere hämatologische Tumorerkrankungen. Hier befinden sich CAR-T-Zellen, die das Antigen BCMA (B-cell Maturation Antigen) erkennen, in fortgeschrittener klinischer Erprobung zur Behandlung des multiplen Myeloms [26]. Danach folgen CD30 (Hodgkin-Lymphom) sowie die B‑Zellmarker CD123, CD22 und CD20 (Abb. 3a). Letztere sind unter anderem als alternative Zielantigene zu CD19 in der Testung. Weil es bei der CAR-Therapie zu antigennegativen (z. B. CD19-negativen) Rückfällen kommen kann [27], sind die Erforschung und Verfügbarkeit mehrerer Marker für das gleiche Ziel von hoher Bedeutung für die Gewährleistung langfristig effektiver Therapie. Erste Studien untersuchen nun auch den Einsatz der CAR-Therapie bei T‑Zelllymphomen, etwa über CD7. Hier müssen allerdings intelligente Maßnahmen ergriffen werden, um einen „Brudermord“ an den ebenfalls CD7-positiven CAR-T-Zellen zu verhindern [28].

Die Bekämpfung von soliden Tumoren stellt einen nächsten Schritt in der Anwendung von CAR-Zellen dar, der sich noch gänzlich in frühen klinischen Phasen befindet. Hier ergeben sich neue Herausforderungen für klinische Forscher, die bereits in dutzenden Studien untersucht werden (Abb. 3b). So haben Studien zur Bekämpfung von Neuroblastomen mit gegen GD2-spezifischen CAR-T-Zellen zwar nur geringe Behandlungserfolge gezeigt, aber Einblicke in die zugrunde liegenden Mechanismen erlaubt. Demnach tragen eine unzureichende Persistenz der CAR-T-Zellen, nicht ausreichend selektive Tumormarker und immunsuppressive Tumormikroumgebungen zur bisher geringen Effektivität bei [29]. Trotz dieser Schwierigkeiten werden ermutigende Fortschritte gemacht: So hat eine klinische Studie zur Behandlung von Pleuramesotheliomen mit mesothelingerichteten CAR-T-Zellen in Kombination mit PD-1-Blockade die Sicherheit der Produktanwendung und erste Hinweise auf Effektivität geliefert [30]. Die CAR2BRAIN-Phase-1-Studie in Frankfurt am Main wird zeigen, ob CAR-NK-Zellen gerichtet auf das Tumorantigen Her2/neu das Potenzial haben, den Herausforderungen der soliden Tumorumgebung im Kontext von Glioblastomen zu begegnen [31]. Die Gesamtzahl von CAR-NK-Studien mit aktuell 15 ist allerdings noch gering [32]. Dennoch werden die Ergebnisse dieser Studien in Fachkreisen mit Spannung erwartet.

## Hürden in der Anwendung und mögliche Lösungsansätze

In den oben zusammengefassten klinischen Anwendungen werden sehr schwer erkrankte Patienten, die beispielsweise an aggressiven, gegenüber etablierten Arzneimitteln resistenten Leukämien oder Lymphomen leiden, mit CAR-T-Zellen behandelt. Die dabei erzielten Behandlungserfolge rechtfertigen die Hoffnung, dass die CAR-Therapie eine breit anwendbare Krebstherapie für viele Patienten werden könnte. Jedoch ist die klinische Anwendung von CAR-T-Zellen nicht ohne Probleme. Können diese adressiert werden, könnte die Nutzen-Risiko-Abwägung auch für Patienten in früheren Stadien der Erkrankung zugunsten der CAR-T-Therapie ausfallen.

### Nebenwirkungen

CRS ist ein Sammelbegriff für ein Spektrum klinischer Symptome und Laborbefunde, die bei mit CAR-T-Zellen behandelten Patienten auftreten. Dazu gehören Fieber, niedriger Blutdruck, Hypoxie und neurologische Auffälligkeiten sowie deutlich erhöhte Level von Zytokinen im Serum. Wenngleich CRS verschiedener Schweregrade nicht nur bei Anti-CD19-CAR-Therapie auftritt, sondern auch im Kontext anderer Zielantigene beschrieben wurde, treten die meisten Fälle schweren Zytokinfreisetzungssyndroms bei der Behandlung hämatologischer Krankheitsbilder mit Anti-CD19-CARs auf [14, 33]. CRS tritt bei mehr als der Hälfte der mit Tisagenlecleucel oder Axicabtagene ciloleucel behandelten Patienten auf. Jedoch ist dabei die Inzidenz schwerer, medikamentöse Intervention erfordernder Fälle von CRS vom Produkt und der Indikation abhängig. So reichte die Frequenz schwerer Fälle in den relevanten Zulassungsstudien von 12 % für Axicabtagene ciloleucel über 22 % für Tisagenlecleucel bei Lymphomen bis zu 44 % für Tisagenlecleucel bei Leukämien [19, 20, 34].

Zusätzlich ist bei der CAR-T-Behandlung von hämatologischen Krankheitsbildern Neurotoxizität beschrieben worden, die für wenige Patienten tödlich war [4, 14]. Zur Milderung schwerer Nebenwirkungen der CAR-T-Therapie werden Patienten mit Corticosteroiden, allein oder in Kombination mit dem antagonistischen Anti-IL6R-Antikörper Tocilizumab, behandelt. Während inzwischen genaue Protokolle für das Management sowohl von CRS als auch (der schlechter handhabbaren) Neurotoxizität existieren [34, 35], ist das molekulare Verständnis der Phänomene noch unzureichend, um für alle Fälle hocheffektive Interventionen zu definieren, die das therapeutische Potenzial der CAR-T-Zellen (im Gegensatz zu Corticosteroidbehandlung) erhalten.

**Figure 4:**
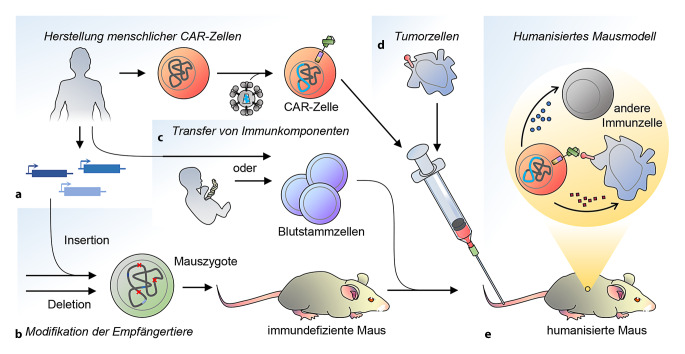

Zur präklinischen Untersuchung von CRS und Neurotoxizität werden häufig Mausmodelle eingesetzt. Weil es gilt, die Wirkung menschlicher CAR-Zellpräparate zu evaluieren, werden dafür komplexe, sogenannte humanisierte Mausmodelle verwendet. Aufgrund der hohen molekularen Spezifität von CAR-Zellen muss ein dem menschlichen möglichst ähnlicher immunologischer Kontext geschaffen werden. Hierbei gilt es nicht nur, Zielzellen mit dem Zielantigen bereitzustellen, sondern auch, Reaktionen des murinen Immunsystems auf menschliche Zellen und – wichtiger – der implantierten menschlichen Immunzellen auf das murine Wirtsgewebe (Transplantat-gegen-Wirt-Reaktion, engl. Graft versus Host Disease, GvHD) zu verhindern bzw. hinauszuzögern, um die Aussagekraft des Modellsystems zu gewährleisten. Wie in Abb. 4 veranschaulicht, werden dazu menschliche Blutstammzellen in durch genetische Manipulation immundefizient gemachte Mäuse transplantiert. In so humanisierten Mäusen können dann die Interaktionen zwischen menschlichen CAR-Zellen und Tumorzellen sowie zwischen CAR-Zellen und anderen Immunzellen charakterisiert werden, um die dem CRS und der Neurotoxizität zugrunde liegenden Mechanismen zu erschließen. So konnten Experimente in mit adulten Blutstammzellen humanisierten NSG-Mäusen die Antitumoreffekte, das CRS und die Neurotoxizität von Anti-CD19-CAR-T-Zellen rekapitulieren. Interessanterweise konnte dabei Monozyten eine wichtige Rolle zugeschrieben werden und neben Interleukin‑6 (IL-6) konnte IL‑1 als Ursache für CRS und Neurotoxizität identifiziert werden [6]. Komplementär dazu sind Ergebnisse aus einem Modell auf Basis immundefizienter Mäuse ohne separate menschliche Immunrekonstitution, die den Ausstoß von IL‑6, IL‑1 und Stickoxid (NO) durch Makrophagen als kritische Regulatoren des CRS identifizierten [5].

Humanisierte Modelle sind hilfreich zur direkten Evaluierung von für die Behandlung von Menschen gemachten Zellpräparaten. Ihre komplexe Herstellung macht sie allerdings fragil in der Handhabung und teuer in der Herstellung. Zudem kann durch die hybride Natur der humanisierten Immunsysteme nicht sichergestellt werden, dass die scheinbar wichtigen Schnittstellen zwischen den myeloiden und lymphoiden Komponenten des Immunsystems korrekt abgebildet sind. Ein weiterer Ansatz, der auch bei der präklinischen Evaluation von Axicabtagene ciloleucel verfolgt wurde [36], ist, ein zum menschlichen System analoges, komplett murines System zu verwenden. Dabei werden murine Antigene erkennende CARs auf murinen Zellen zur Bekämpfung von Tumorzellen murinen Ursprungs eingesetzt. Eine Angleichung an die klinische Situation kann etwa durch Bestrahlung der Mäuse oder Vorbehandlung mit Chemotherapeutika erfolgen. Letztere Strategie wurde verwendet, um den Einfluss von Katecholaminsignaling auf CRS in einem syngenen Mausmodell zu untersuchen [8].

Die der Neurotoxizität zugrunde liegenden Mechanismen sind unzureichend verstanden. Allerdings demonstrierte eine klinische Studie vor Kurzem, dass die Verwendung eines CAR mit ausschließlich menschlichen Sequenzen (im Gegensatz z. B. zu den in Axicabtagene ciloleucel und Tisagenlecleucel verwendeten, von der Maus abgeleiteten Antigenbindedomänen) in wesentlich geringeren Raten in Neurotoxizität resultierte [37]. Eine weitere Möglichkeit, adverse Effekte zu umgehen oder zu mindern, könnte in der Verwendung von CAR-NK-Zellen bestehen. Es ist – vermutlich wegen ihrer von T‑Zellen abweichenden Aktivierungsmechanismen – möglich, allogene NK-Zellen sicher adoptiv zu transferieren [16]. Eine neue klinische Phase-1/2-Studie zum Einsatz von Anti-CD19-CAR-NK-Zellen bei B‑Zelltumoren fand keine Belege für CRS, Neurotoxizität oder GvHD bei der Transplantation partiell MHC-inkongruenter und/oder killer-immunoglobulin-like-receptor-(KIR-)inkompatibler allogener CAR-NK-Zellen [38]. Zur eindeutigeren Beurteilung der Antitumoraktivität von CAR-NK-Zellen bedarf es allerdings weiterer Studien.

### Herstellung

Eine zweite wesentliche Hürde für die breite klinische Anwendung von CAR-Zellen stellt die logistisch sehr aufwendige, wenig standardisierte, fehleranfällige und teure Herstellung dar. Aktuell werden CAR-T-Zellprodukte autolog, d. h. aus der Leukapherese von Patienten hergestellt. Dies verlangt nach gut an die jeweiligen Behandlungszentren angeschlossener Good-Manufacturing-Practice-(GMP-)Infrastruktur mit Kapazitäten für individualisierte Bioprozesstechnik. Wohl auch als Folge von Faktoren wie der logistischen und technischen Schwierigkeit der Herstellung des Zellprodukts sind die Kosten für eine Behandlung mit kommerziellen CAR-T-Produkten prohibitiv hoch: Gemäß den Beschlüssen des Gemeinsamen Bundesauschusses zu Tisagenlecleucel und Axicabtagene ciloleucel liegen die Jahrestherapiekosten pro Patient bei ca. 320.000 € bzw. ca. 390.000 € [39, 40]. Abhängig vom Therapiezentrum können sich die Jahrestherapiekosten basierend auf der krankenhausindividuellen Vereinbarung der Vergütung von neuen Untersuchungs- und Behandlungsmethoden (NUB) jedoch auch unter 300.000 € belaufen [41]. Durch einen Mangel an Standardisierung existiert eine erhebliche Heterogenität unter den Verfahren, die weltweit zur Produktion autologer CAR-T-Produkte eingesetzt werden.

Gemeinsam ist den Verfahren hohe Variabilität innerhalb des Prozesses, zum Beispiel betreffend Zellzahlen, Zellwachstum und Transduktionseffizienz. Die „offene“ Manipulation der Zellen im Verlauf des Prozesses macht diesen anfällig für Kontamination und andere Handhabungsfehler. Für viele Schritte, zum Beispiel die Zellisolierung, die Aktivierung der Zellen oder die Transduktion, ist unklar, welche Technik optimale Ergebnisse erzielt [17, 18]. Ein wichtiger Schritt hin zu einer konsistenteren (und damit kosteneffektiveren) Herstellung von CAR-T-Zellen könnte die Automatisierung des Herstellungsprozesses sein. Mit ihr könnten die mit manuellen Arbeitsschritten verbundenen Risiken und Variationen eliminiert werden. Während automatisierte Zellkulturplattformen der ersten Generation – zur Minimierung der von manuellen Schritten ausgehenden Prozessvariabilität – einzelne Abschnitte eines Bioprozesses automatisieren, können automatisierte Zellkulturplattformen der zweiten Generation als Zusammenfassung eines ganzen personalisierten Bioprozesses in einem Gerät verstanden werden, die vollautomatische Produktion z. B. eines CAR-T-Zellprodukts ermöglichen [42].

Die durchschnittliche Herstellungsdauer von autologen CAR-T-Zellprodukten beträgt 12 Tage (7–22 Tage; [17]). Bei aggressiven Krankheitsbildern wäre allerdings die schnellstmögliche Behandlung im Sinne des Patienten, auch weil größere Tumorlast im Knochenmark mit schwererem CRS assoziiert wurde [34]. Zudem können der schlechte Zustand des Patienten zum Zeitpunkt der Spende und der damit assoziierte suboptimale Zustand der Leukapherese hinsichtlich Viabilität und Zellzahlen die Produktion des CAR-T-Zellprodukts erheblich erschweren. Zusätzlich besteht bei Zellspenden aus leukämischen Patienten das Risiko der Transduktion und Reinfusion leukämischer Blasten. Ein solcher Fall von CAR-Blasten mit tödlichem Ausgang ist aus einer Phase-1-Studie zu Tisagenlecleucel bekannt [43]. Die Verwendung allogener, d. h. von gesunden Spendern abgeleiteter CAR-T-Zellen könnte diese Probleme umgehen helfen. Allogene CAR-T-Zellen würden auf Basis „fitterer“ Zellen produziert und wären damit konsistenter und einfacher herzustellen. Sie würden sich im Vornherein herstellen lassen und wären zum klinisch sinnvollsten Zeitpunkt einsetzbar, wenn nötig mit minimaler Verzögerung zwischen Behandlungsentscheidung und Behandlung.

Ähnlich wie bei der Spende von Blutstammzellen [44] gilt es allerdings, das Risiko durch Implantation fremder Immunzellen ausgelöster Komplikationen einzuschätzen. Zu diesen zählen GvHD und durch den Wirt vermittelte Abstoßung der transplantierten Zellen (engl. Host-mediated Rejection). Strategien zur Kontrolle dieser Effekte beinhalten die genetische Ablation der Expression von T‑Zellrezeptoren (TCRs) und MHC-Molekülen auf Spenderzellen. Des Weiteren können Spenderzellen (z. B. durch die Entfernung von CD52) so modifiziert werden, dass sie – im Gegensatz zu Wirtsimmunzellen – resistent gegenüber Lymphodepletion sind. Entsprechende klinische Studien laufen [45].

Ebenfalls könnten nichtvirale Transferstrategien dazu beitragen, die Produktion von CAR-Zellen *ex vivo* zu verbessern. CAR-Transgene können z. B. als Plasmide mittels Elektroporation in die Immunzelle eingebracht und mittels Transposasen in deren Genom integriert werden oder in Form von synthetischer mRNA transferiert werden, die eine zeitlich begrenzte CAR-Expression ermöglicht. Vorteile gegenüber viralem Transfer sind dabei niedrigere Kosten und ein geringeres Risiko von insertioneller Onkogenese [18]. Gegenstand aktueller Forschung ist ebenfalls die Verwendung von sogenannten Genscheren wie der CRISPR/Cas9-Technologie, um eine präzise Veränderung des Immunzellgenoms zu erreichen, z. B. die gezielte Insertion des CAR-Gens in einen T‑Zellrezeptorlokus [46].

Zukünftig könnte ein alternativer Ansatz in der Generierung von CAR-T-Zellen *in vivo* liegen, also direkt im Patienten. Dafür müssten lediglich Vektoren injiziert werden, welche die für den CAR codierende Genkassette spezifisch in die T‑Zellen einbringen. Diese Vorgehensweise befindet sich allerdings noch in der präklinischen Entwicklung. In einem Ansatz wurden mithilfe von CD3-targetierten Nanopartikeln CAR-T-Zellen in Mäusen generiert [47]. In einer weiteren Arbeit konnten humane CAR-T-Zellen mit CD8-targetierten lentiviralen Vektoren in humanisierten Mäusen erzeugt werden [7, 48]. Ob solche *In-vivo*-Ansätze tatsächlich das Potenzial, haben die komplexe *Ex-vivo*-Herstellung von CAR-T-Zellen zu umgehen, wird sich an Großtiermodellen erweisen müssen.

## Fazit und Ausblick

Mit der CAR-Therapie beginnt ein neues Kapitel der Krebstherapie. Trotz der rapiden Entwicklungen in der Erforschung und Anwendung von CAR-Therapien und ihrer beeindruckenden Erfolge ist Geduld angebracht. CAR-Produkte gehören zur bisher kleinen Gruppe der Arzneimittel für fortgeschrittene Therapien (engl. Advanced Therapy Medicinal Products, ATMPs) und sind damit einzigartig unter den Krebstherapien. Dementsprechend klein ist die Erfahrungsbasis, nicht nur für die hier besprochenen technischen Aspekte ihrer Herstellung und Anwendung, sondern auch für ihre Zulassung durch die entsprechenden regulatorischen Institutionen. Das durch die Studienlage unterstrichene globale Interesse, CAR-T-Therapien voranzubringen und breit nutzbar zu machen, bereitet den Boden für die Erforschung, Zulassung und Anwendung von CAR-ATMPs nicht nur für weit fortgeschrittene Tumorerkrankungen, sondern auch für eine Bandbreite von anderen Indikationen, von Infektionskrankheiten wie HIV [49] über Autoimmunkrankheiten bis hin zur allogenen Transplantation [50]. So könnte die Krebstherapie mit chimären Antigenrezeptoren als Türöffner für neue Entwicklungen in der Zelltherapie fungieren, die das Potenzial haben, nicht nur die Onkologie nachhaltig zu verändern.
